# Editorial: Radiological Emergencies and the Medical Physicist

**DOI:** 10.1120/jacmp.v13i1.3798

**Published:** 2012-01-05

The events at the Fukushima Daiichi nuclear power plant in Japan prompted an editorial^(^ ^1^ ^)^ written by Bill Hendee, PhD, Editor‐in‐Chief of *Medical Physics*, in which he discussed the role of medical physicists as potential volunteers in responding to public health emergencies, especially radiological emergencies of various types. In order to obtain more widespread dissemination of this information, the JACMP, along with several other medical physics journals, is reprinting Dr. Hendee's editorial. Comments, of course, are most welcome.

George Starkschall, PhD

Editor‐in‐Chief

January 5, 2012

## Footnotes

1 Med Phys. 2011;38:2311–12. (Reprinted with permission of the author)

